# Meeting report: GSC M5 roundtable at the 13th International Society for Microbial Ecology meeting in Seattle, WA, USA August 22-27, 2010

**DOI:** 10.4056/sigs.1333437

**Published:** 2010-12-15

**Authors:** Jack A. Gilbert, Folker Meyer, Rob Knight, Dawn Field, Nikos Kyrpides, Pelin Yilmaz, John Wooley

**Affiliations:** 1 Argonne National Laboratory, 9700 South Cass Avenue, Argonne, IL 60439, USA.; 2 Department of Ecology and Evolution, University of Chicago, 5640 South Ellis Avenue, Chicago, IL 60637, USA.; 3 Computation Institute, University of Chicago, 5640 South Ellis Avenue, Chicago, IL 60637, USA.; 4 Department of Chemistry and Biochemistry, University of Colorado, Boulder, CO 80309, USA; 5 NERC Centre for Ecology & Hydrology, Crowmarsh Gifford, Wallingford, Oxford, OX10 8BB, UK.; 6 DOE Joint Genome Institute, Walnut Creek, CA 94598, USA; 7 Microbial Genomics Group, Max Planck Institute for Marine Microbiology, D-28359 Bremen, Germany; 8 University of California San Diego, 9500 Gilman Drive, La Jolla, CA 92093, USA

## Abstract

This report summarizes the proceedings of the *Metagenomics, Metadata, Metaanalysis, Models and Metainfrastructure* (M5) Roundtable at the 13th International Society for Microbial Ecology Meeting in Seattle, WA, USA August 22-27, 2010. The Genomic Standards Consortium (GSC) hosted this meeting as a community engagement exercise to describe the GSC to the microbial ecology community during this important international meeting. The roundtable included five talks given by members of the GSC, and was followed by audience participation in the form of a roundtable discussion. This report summarizes this event. Further information on the GSC and its range of activities can be found at http://www.gensc.org.

## Introduction

Research in Microbial Ecology has entered a brave new world. For a few hundred years we have adequately understood the concept of microbial life; however, we have lacked the tools to explore that life in detail. Using microscopes and culturing techniques, we have managed to describe approximately 9-10 thousand species of the *Bacteria*, and for a much smaller number of the *Archaea*. Yet, by isolating microbial DNA from different ecosystems, we have demonstrated that these cultured representatives barely describe 30% of the phylum-level phylogenetic diversity that exists. Indeed, cultured isolates may represent less than 5% of the total microbial diversity in any given environment [1].

In terms of investigating the microbiome of a sample, (e.g. soil, marine, human-associated) it is the numbers of bacteria involved that are truly staggering. It is estimated that there are more than 1 nonillion microbial cells on planet earth (1 x 10^30^), with around 100 trillion existing on or in every human being. With this vast array of life comes potentially indescribable diversity. In part, this diversity is driven by the length of time (approximately, 3.8 billion years) these organisms have had to evolve. However, the rate at which they evolve must also play a significant role. The species concept so important for our understanding of eukaryotic systems takes a lesser role and may be less well defined in *Bacteria* and *Archaea*. The so-called pan-genome concept [2] attempts to describe the genetic information shared by and overall unique to members of a given taxonomic grouping.   The concept partially describes how these groups exchange and utilize genetic information to exploit the vast diversity of environmental niches (both temporal and spatial) in an ecosystem.

As the research problem of describing microbial life on earth and thereby, devising new ecological theory to explain how it all works is so large, the microbial ecology community must advance how it produces and manages its sample and derived data information to provide a framework for improved collaboration and understanding. The global public repositories contain approximately 500 billion base pairs of genetic information isolated and sequenced from microbial communities around the world. Yet, this constitutes only 1/8^th^ or ~12% of the genetic information found in a milliliter of sea water or a gram of soil. This hardly constitutes the kind of coverage or depth required to explore the basic principles governing microbial ecology. However, while the rapid, sustained provision of ever more sequences will help us to explore more of the genetic diversity in an ecosystem, it will not provide us with greater ability to explain ecological theory without the development of an integrative approach for analysis.

Unlike eukaryotic ecology, which until recently was nearly entirely reliant on morphological characteristics and the relative abundance of species, microbial ecology (encompassing bacterial, archaeal, eukaryal and viral) is just learning how to explain functional and phylogenetic diversity appropriately. Strikingly, the more we uncover the more we have to adjust our metrics to cope. Whereas the phenotypic characteristics of eukaryotic macrofauna and macroflora enable relatively simple comparative analysis to be made among multiple projects, the same cannot be said for microbial ecology. The lack of understanding in how we delineate species or functions has led to a considerable gap in our ability for comparison across microbial projects.   It is therefore essential that we work as a community to explore ways of rectifying this.

There are currently some 8,000 metagenomic projects available. However, as these have been run on different sequencing platforms, with different amplification strategies, different PCR primers, and different sampling regimens it is virtually impossible to make any comparisons between them.   In addition, very few projects are submitted to public repositories with any physical and chemical parameter data, which could help to describe the environment from which the samples were isolated.  Comprehensive metadata, which could be used to establish which projects can be more usefully compared, is generally not available; this lack of contextual information significantly compounds the difficulties.  Specifically, the metadata needs to include at least the minimum information that would enable us to explore ecological metrics among samples.  This is essential if we are to start exploring the factors that shape microbial ecology, which directly or indirectly will therefore influence all biochemical and climatic events on the planet. Doing so would allow us to turn the data from metagenomics research into ecological knowledge.

There are currently three big challenges to overcome for microbial ecologists. Firstly, we must understand how to explore systems biology, community interactions and modeling, i.e. we need to know what parameters are required. Secondly, we must explore how to use these metrics and models to explore predictive biology, in much the same way as we physical scientists make predictions and extend the understanding of  physical world.    Third, only through deep exploration of natural microbial communities will we discover the "Dark Matter" of the microbial world and achieve the full potential for societal applications of novel microbial biology and biochemistry.

The M5 roundtable aimed to introduce the Genomic Standards Consortium to the microbial ecology community at the International Symposium on Microbial Ecology, 13^th^ Meeting and promote increased future engagement. The Genomic Standards Consortium exists to improve the utility of public collections of completed genomes and metagenomes, through the development and dissemination of standards and data integration technologies. The GSC is an open-member international community consisting of biologists, bioinformaticians and computer scientists that includes representatives from EMBL, EMBL-EBI, DDBJ, NCBI, and major sequencing centers including JCVI, JGI, and the Wellcome Trust Sanger Institute (WTSI). Core work of the GSC includes the development and implementation of minimal information checklists. The first major accomplishment of the GSC was the publication of the ‘Minimum Information about a (Meta) Genome Sequence’ (MIGS/MIMS) specification [3], which describes the core information that should be reported with each new genome or metagenome publication and has been supported by major data producers/brokers (such as GOLD [4], MG-RAST [5] and CAMERA [6]), and is supported by the archival sequence resources of EBI, DDBJ and NCBI. More recently, the GSC launched this open-access, online journal to provide a forum for presentation of standards-compliant genome reports, standard operating procedures and other related articles. Further GSC projects include the Genomic Contextual Data Markup Language (an XML data format to support minimal standards [7] ), the Genomic Rosetta Stone (a resolving service for top-level genome and metagenome project information from different resources [8]; ) and Habitat Lite (a lightweight ecological ontology [9] ). Along with individual grants, the GSC currently receives support from the US National Science Foundation (NSF) under a Research Coordination Network grant awarded in 2009. Membership and community awareness of GSC has risen, such that meetings have grown in size substantially over the history of the GSC. GSC 11 and GSC 12 are already being planned for 2011 at the Wellcome Trust Genome Campus in Cambridge England and the Max-Plank Institute for Marine Microbiology in Bremen Germany, respectively.

The topics presented and discussed at this roundtable were broad and focused on the value of richer stores of metadata associated with public data and ongoing GSC projects to improve metadata capture and exchange. The ultimate goal of the roundtable was to attract experimentalists in microbial ecology to appreciate and review  the recommendations of the GSC,  understand the rationale behind the development of new standards, and ultimately, participate in their usage  We, therefore, divided the roundtable into two parts, beginning with a set of five talks and following with an open forum in which the community could ask questions regarding GSC activities.

## Talks

**Rob Knight (University of Colorado)** provided numerous examples of the power of having metadata associated with environmental and host-associated 16S rRNA amplicon datasets. This umbrella talk helped to explore the ramifications of data standardization and potential routes for implementation. Importantly, this talk was from an experimentalist’s perspective and helped to drive forward the concepts from that standpoint. **Dawn Field (Centre for Ecology and Hydrology)**, the president of the GSC, described the history of the organization and gave an overview of all of the current organizational efforts. **Jack Gilbert (Argonne National Laboratory)** provided an overview of a new mega sequencing initiative, the Earth Microbiome Project [10], which aims to catalogue global functional and phylogenetic diversity through the sequencing of ~200,000 environmental samples; he also explored the equally vital Microbial Earth Project [11], which aims to close the genomes of 10,000 cultured *Bacterial* and *Archaeal* type strains from culture collections around the world. Both of these projects highlighted the vast information flux, which is become real for microbial ecology. With vast data come great responsibility; it is essential that these projects are made available for the whole community, and as such, it is vital that appropriate reporting standards are implanted.

**Folker Meyer (Argonne National Laboratory)** explored the computational requirements for coping with the data wave. The fast growth of data will require the various analysis providers to not only collaborate in order to avoid duplication of effort but also improve the computational cost of their various analysis steps dramatically. The M5 group (metagenomes, metadata, metamodels, metainfrastructure and metanalysis) is working together to develop the standards required for sharing data and computational results in a democratized sequencing world, standards that will add transparency to and enable sharing among the providers of analysis like IMG/M [12], MG-RAST [5] and CAMERA [6]. Standard formats for data and result sharing will require machine-readable descriptions of computational results (metadata on bioinformatics analysis). Components of this format will be the MIMS information in GCDML and descriptions in abstract workflow formats. **Pelin Yilmaz (MPI-Bremen)**, explained the  newest GSC minimum information checklist and how it should be used. The objective of the “Minimum Information about an Environmental Sequence” (MIENS) checklist is to aid in the description of phylogenetic and functional marker genes from all three domains of life, from surveys to cultured organisms and independent of the sequencing platform used. The talk highlighted the pressing need for better contextual metadata standards for marker genes in the age of mass-sequencing in microbial ecology, and briefly showed the GSC roadmap to tackle with this issue with the outcome as MIENS. The core standard was presented, with a focus on examples of compliant datasets and submission of compliant data to INSDC databases. The audience was encouraged to give feedback and participate in the further development and applications of MIENS.

## Panel and Roundtable Discussion

A wide disciplinary range of microbial ecologists participated in the roundtable, which was well attended (approximately 60-70 attendees), especially considering the many roundtables running in parallel.  A lively discussion ensued and a sense that the work of the GSC had a growing place in the world of microbial ecology was established. There was discussion about the practicalities of complying with the GSC family of standards and an emphasis on the fact that it was the responsibility of the GSC to make compliance as easy as possible for experimentalists. This would need to be achieved through documentation, development of tools and re-engineering of the core databases in this field. It was agreed that the vision of a future store of richly annotated genomes, metagenomes and marker sequences was worth working for, but would need both community buy-in and engagement as well as dedicated funding.

## Conclusions

Being chosen for an opportunity for the GSC to host a roundtable at ISME13, especially given such fierce competition for these fora, demonstrates that the microbial ecology community understands the importance of data standards in recording and interpreting biological information. Importantly, the community further understands that when working towards descriptive and predictive models that will require data generation from multiple sources, this endeavor is imperative.  The GSC looks forward to future opportunities in engaging the experimental community and advancing the potential of microbial ecology.
